# Conformational Switch Regulates the DNA Cytosine Deaminase Activity of Human APOBEC3B

**DOI:** 10.1038/s41598-017-17694-3

**Published:** 2017-12-12

**Authors:** Ke Shi, Özlem Demir, Michael A. Carpenter, Jeff Wagner, Kayo Kurahashi, Reuben S. Harris, Rommie E. Amaro, Hideki Aihara

**Affiliations:** 1 Department of Biochemistry, Molecular Biology and Biophysics, University of Minnesota, Minneapolis, Minnesota 55455 USA; 2 Masonic Cancer Center, University of Minnesota, Minneapolis, Minnesota 55455 USA; 3 Institute for Molecular Virology, University of Minnesota, Minneapolis, Minnesota 55455 USA; 4 Department of Chemistry and Biochemistry, University of California, San Diego, La Jolla, CA 92093 USA; 5 Center for Genome Engineering, University of Minnesota, Minneapolis, Minnesota 55455 USA; 6 Howard Hughes Medical Institute, University of Minnesota, Minneapolis, Minnesota 55455 USA

## Abstract

The APOBEC3B (A3B) single-stranded DNA (ssDNA) cytosine deaminase has important roles in innate immunity but is also a major endogenous source of mutations in cancer. Previous structural studies showed that the C-terminal catalytic domain of human A3B has a tightly closed active site, and rearrangement of the surrounding loops is required for binding to substrate ssDNA. Here we report structures of the A3B catalytic domain in a new crystal form that show alternative, yet still closed, conformations of active site loops. All-atom molecular dynamics simulations support the dynamic behavior of active site loops and recapitulate the distinct modes of interactions that maintain a closed active site. Replacing segments of A3B loop 1 to mimic the more potent cytoplasmic deaminase APOBEC3A leads to elevated ssDNA deaminase activity, likely by facilitating opening of the active site. These data collectively suggest that conformational equilibrium of the A3B active site loops, skewed toward being closed, controls enzymatic activity by regulating binding to ssDNA substrates.

## Introduction

The APOBEC3 family of single-stranded DNA (ssDNA) cytosine deaminases plays important roles in innate immunity against viruses and transposons (reviewed by refs^1–3^). However, a subset of APOBEC3 proteins, most notably the nuclear-localized APOBEC3B (A3B) enzyme, is strongly implicated in causing mutations in various human cancers (reviewed by refs^3–6^). Consistent with a major role in tumor evolution, A3B overexpression has been linked to poorer prognosis for patients with multiple cancer types^7–13^.

Given the importance of A3B-mediated mutagenesis and its potential as a target of anticancer therapy, we are interested in understanding the molecular mechanisms of ssDNA deamination by A3B and its regulation through structural analyses. Human A3B consists of tandem zinc-coordinating cytosine deaminase domains: an N-terminal domain with a pseudo active site and a C-terminal functional catalytic domain. The C-terminal domain (ctd) alone is sufficient to function as an active ssDNA deaminase both *in vivo* and *in vitro*, whereas the N-terminal domain may serve to provide additional DNA contacts and/or regulate catalytic activity^14–19^. The N-terminal domain is also responsible for nuclear localization^20–24^.

**Figure 1:**
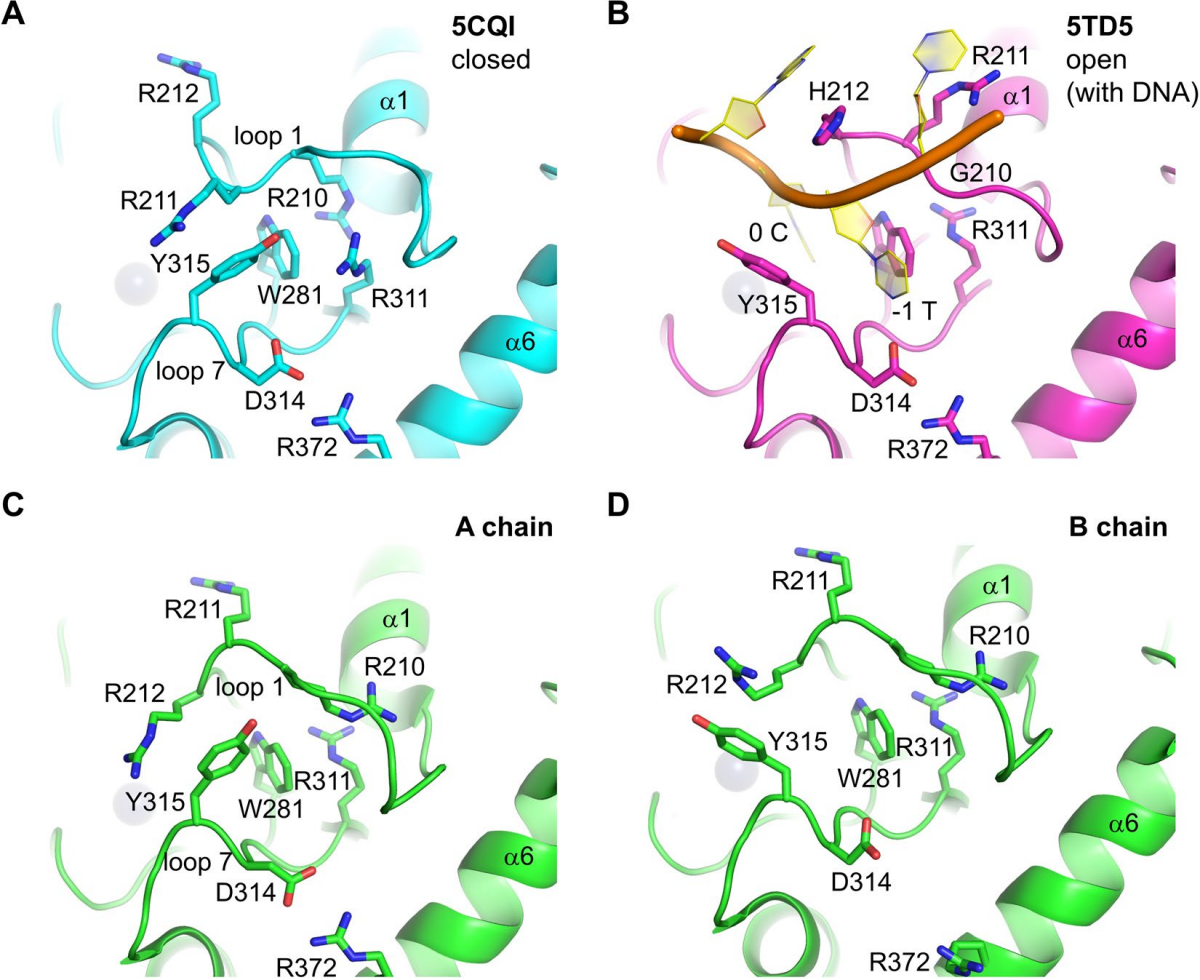
Multiple active site conformations in A3Bctd crystal structures. (**A**) A tightly closed conformation as observed previously in the crystal structure of A3B(187–378)QM_Δloop3^15^. (**B**) The fully open conformation as observed in the crystal structure of an A3Bctd variant bound to ssDNA^25^. (**C**) A-chain of A3B(187–378)QM_Δloop3 in the new crystal form. (**D**) B-chain of A3B(187–378)QM_Δloop3 in the new crystal form. The gray spheres represent the zinc ion in the active site.

**Figure 2:**
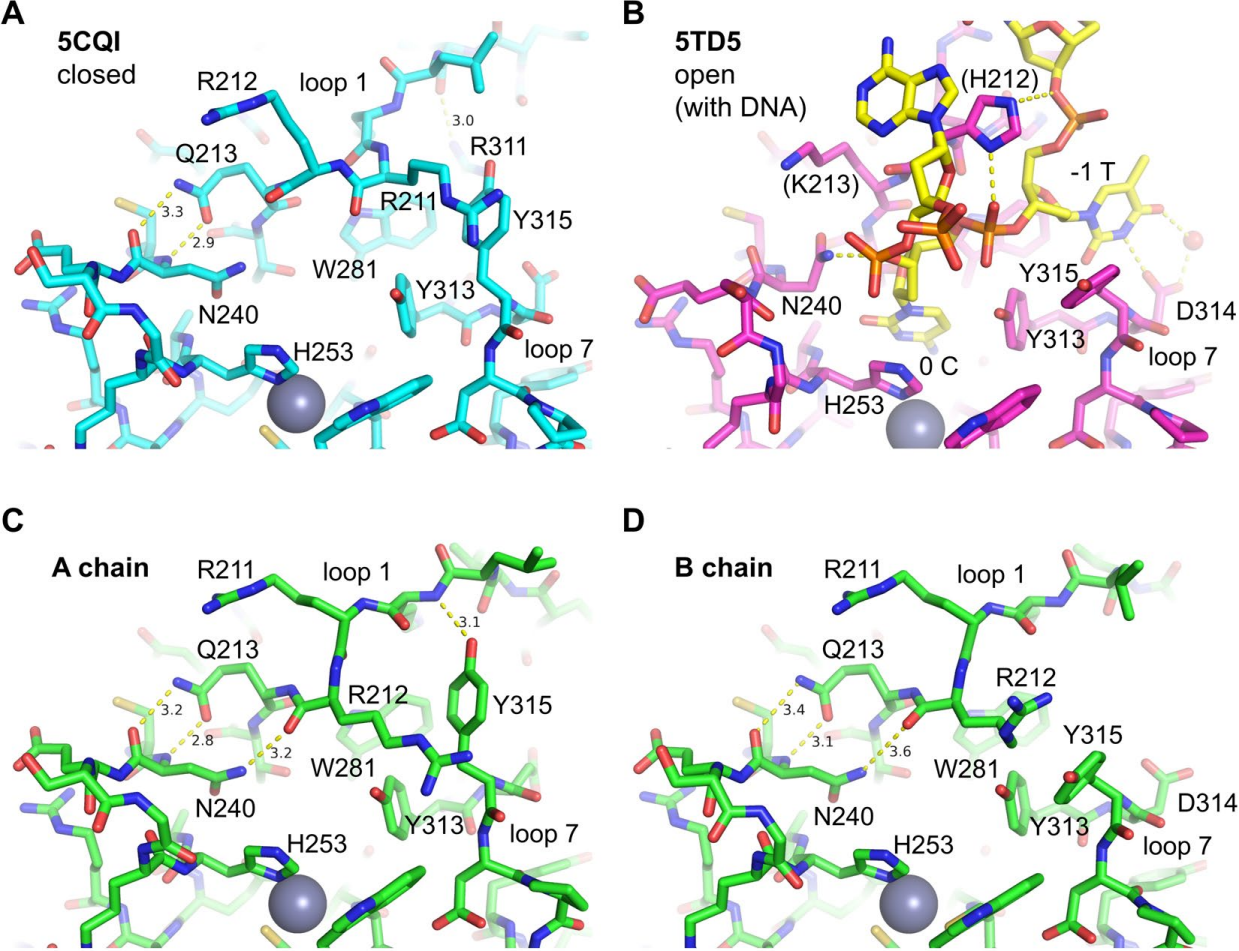
Detailed interactions in the various active site conformations shown in Fig. 1. In all DNA-free structures of A3Bctd (**A**,**C**,**D**), Gln213 at the C-terminal end of loop 1 is hydrogen-bonded to the Asn240 main chain. This positioning of Gln213 is not compatible, for steric reasons, with the conformation of Asn240 observed in the ssDNA-bound A3Bctd (**B**). The δ2 nitrogen atom of Asn240 makes a DNA backbone interaction in the DNA complex. The Asn240 side chain is instead interacting with Arg212 backbone in the new apo crystal structures (**C**,**D**). Tyr315 is in the closed (*gauche* ^+^) conformation in (**A**,**C**) whereas it is in the open (*trans*) conformation in (**B**,**D**). In (**B**), the loop 1 residues including His212 are APOBEC3A-derived. The yellow dotted lines denote hydrogen bonds.

The first crystal structures of A3Bctd showed that it has the canonical zinc-dependent deaminase fold consisting of a central single layer of β-sheet surrounded by α-helices^15^, similar to other APOBEC3 members structurally characterized to date (reviewed by ref.^19^). The active site has a catalytically essential zinc ion coordinated by two cysteines (Cys284 and Cys289) and a histidine (His253), with the fourth position of tetrahedral coordination available for directly interacting with the transition state of the hydrolytic cytosine deamination reaction. The active site pocket is surrounded by three loops (loop 1, 3, and 7). Curiously, A3Bctd crystal structures have shown a closed conformation of the active site loops; Arg211 from loop 1 and Tyr315 from loop 7 are stacked on each other to stabilize the “collapsed” conformations of these loops, limiting accessibility of the active site (Figs 1A and 2A). The tightly closed A3Bctd structure suggested that the active site loops would have to undergo conformational changes to accommodate a ssDNA substrate. The recently solved crystal structure of an A3Bctd variant in complex with ssDNA indeed showed rearrangement of loops 1 and 7, which opens up the active site and generates a narrow groove to accommodate the ssDNA substrate^25^ (Figs 1B and 2B). However, these apo (DNA-free) and ssDNA-bound A3Bctd structures have differed with respect to loop 1 amino acids, which were wild-type and A3A-derived, respectively^15,25^. Thus, our understanding of the conformational flexibility of these active site loops in A3B and potential paths from closed to open states to accommodate ssDNA substrates are still limited.

**Figure 3:**
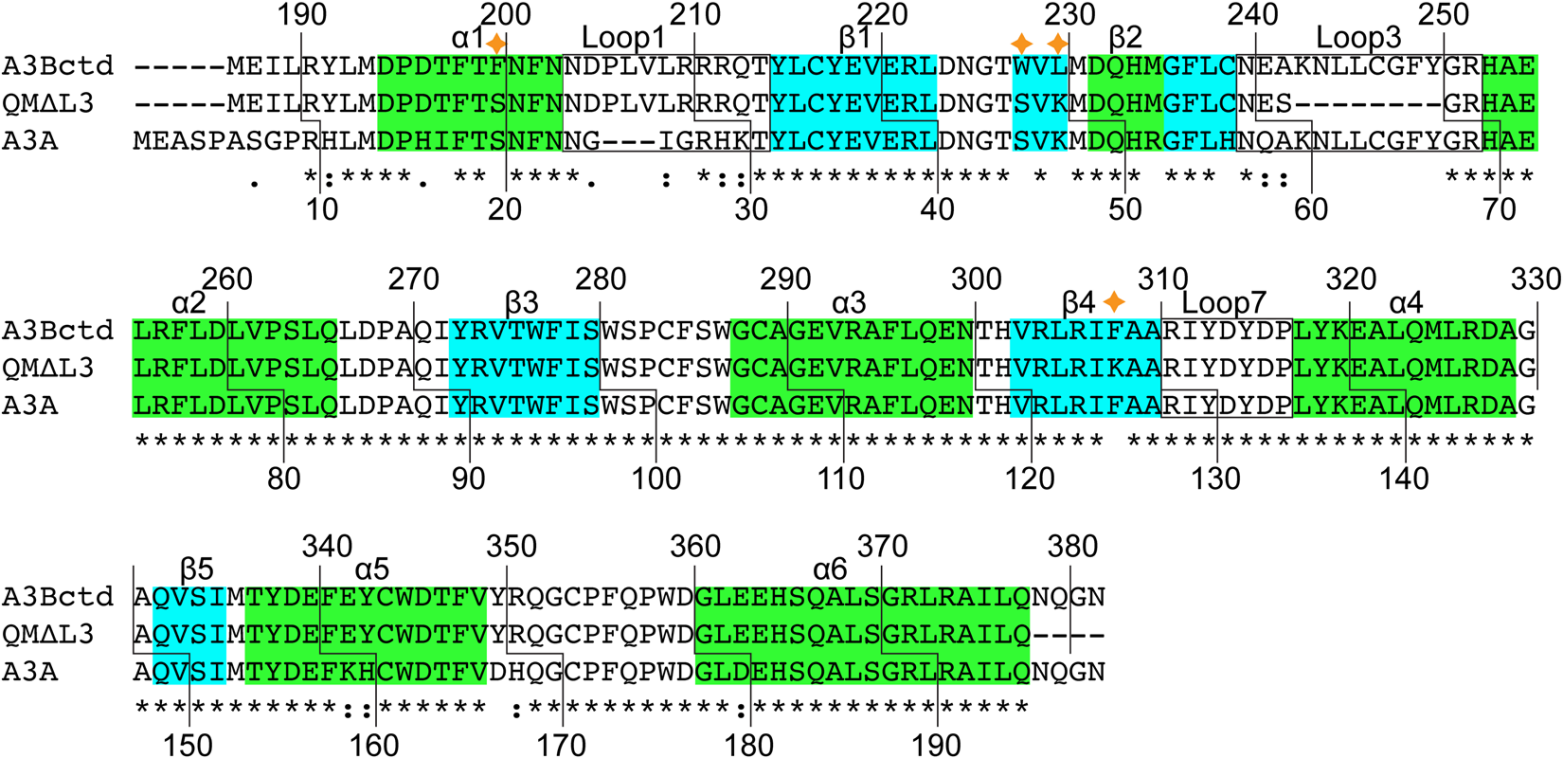
Amino acid sequence alignment comparing wild-type A3Bctd, crystallized A3B(187–378)QM_Δloop3, and wild-type A3A. The residue numbers correspond to those in the wild-type, full-length proteins. Four residues mutated in A3B(187–378)QM_Δloop3 are marked by the stars. The GenBank accession numbers for A3B and A3A are NP_004891.4 and NP_663745.1, respectively.

A3Bctd is closely related (92% amino acid identity) to A3A, which is a single-domain enzyme expressed predominantly in myeloid lineage cells and possesses even greater catalytic activity^26–29^. Whereas most of the structural and catalytic residues are conserved between these enzymes, the amino acid sequences of loop 1 differ significantly (Fig. 3). As loop 1 residues play critical roles in binding ssDNA, the higher deaminase activity of A3A is likely conferred by residues within this loop, including His29 that makes dual phosphate contacts to clamp down the target deoxycytidine nucleotide in the active site^25,30^ (Figs 1B and 2B)^25,30^. These structural insights are supported by biochemical data using wild-type and mutant enzymes^14,16,25,31,32^. In addition, the smaller length of loop 1 of A3A, containing three fewer amino acids than A3Bctd loop 1, may also be important for higher activity due to permitting increased active site accessibility.

To better understand how A3Bctd transitions from a closed state to an open active state and how this process may be regulated by loop 1 and other residues, we pursued additional x-ray structural information for A3Bctd. We also performed multi-copy microsecond molecular dynamics (MD) simulations to examine dynamics of the wild-type A3Bctd active site in the apo form. Similar wild-type A3A MD simulations were done for comparison. In addition, key structural and computational observations were tested by biochemical studies comparing different mutant variants. Overall, our data suggest that A3Bctd catalytic activity is regulated by several distinct conformational switches that govern the transition from a closed, inactive state to an open, active conformation.

## Results

### A3Bctd structure in a new crystal form

**Figure 4:**
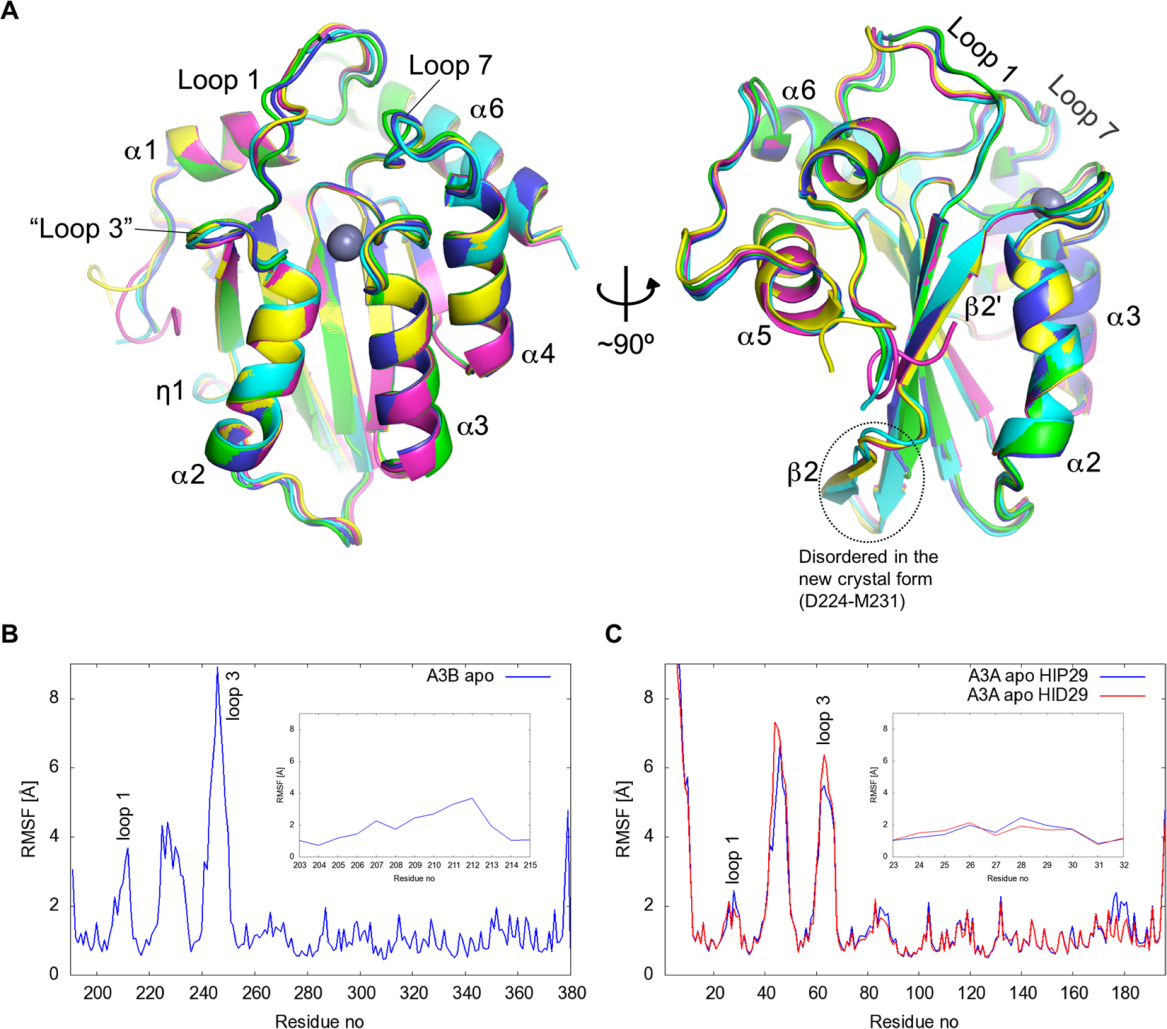
Overall structure of A3Bctd in various crystal forms and flexible regions identified through MD simulations. (**A**) Superposition of all crystallographically independent A3B(187–378)QM_Δloop3 molecules, highlighting high overall similarity as well as significant variation in loop 1 conformations. The structures shown are PDB ID: 5CQD (magenta and yellow), 5CQH (cyan), A-chain in the new crystal form (green), B-chain in the new crystal form (blue). (**B**,**C**) Root-mean-square-fluctuation (RMSF) of non-hydrogen atoms of residues in wild-type A3Bctd (**B**) and A3A (**C**) in MD simulations. Loop 1 residues are shown in detail in the insets.

Using an affinity tag-free construct of the monomeric A3Bctd derivative A3B(187–378)QM_Δloop3, which contains 4 amino acid substitutions (F200S, W228S, L230K, F308K) and a truncation of the flexible loop 3 to improve protein solubility and stability, we obtained a new crystal form of A3Bctd and solved its structure at 1.78 and 1.93 Å resolution (Figs 1C,D, 2C,D, and Table 1; the two datasets show minor structural differences as discussed below). The crystals have two protein monomers in the asymmetric unit, each containing a zinc ion bound in the active site. The two molecules (hereafter referred to as A and B chains) are similar to each other (r.m.s.d. of 0.3 Å for all backbone atoms) and to our previously reported C-terminally His-tagged A3B(187–378)QM_Δloop3 structures (PDB ID: 5CQH, 5CQI, 5CQK, 5CQD) with backbone r.m.s.d. of 0.2 and 0.3 Å, respectively. A superposition of all available A3B(187–378)QM_Δloop3 structures (this study and ref.^15^) shows that the most significant deviation occurs in the loop 1 region, which spans residues Asp205 to Gln213 flanking the active site (Fig. 4A and discussed in greater detail below). In addition, both A and B chains in the new crystal form lack clear electron density for Asp224 to Met231 suggesting disordering of these residues. Further consistent with high local flexibility, this segment, corresponding to the loop following β1 and the first half of the discontinuous β-strand (β2), showed the highest B-factors out of the whole molecule in previously determined A3B(187–378)QM_Δloop3 structures^15^.

**Table 1:** Data collection and model refinement statistics.

PDB ID	A3B/Propandiol	A3B/Ethlene glycol
	5SXG	5SXH
**Data Collection**		
Resolution range (Å)	34.28–1.93 (2.00–1.93)	42.21–1.78 (1.84–1.78)
Space group	P2_1_	P2_1_
Unit cell		
a, b, c (Å)	44.35, 50.7, 76.78	44.31, 50.9, 77.22
α, β, γ (°)	90, 102.86, 90	90, 102.00, 90
Total reflections	81888 (8087)	120529 (11866)
Unique reflections	24909 (2477)	32024 (3182)
Multiplicity	3.3 (3.3)	3.8 (3.8)
Completeness (%)	99.24 (99.16)	98.87 (99.13)
I/σ(I)	10.3 (1.4)	8.8 (1.1)
R-merge (%)	13.52 (101.2)	12.24 (125.0)
R-meas (%)	16.27 (121.1)	14.27 (145.7)
R-p.i.m. (%)	8.93 (65.6)	7.22 (73.6)
CC_1/2_	0.991 (0.547)	0.996 (0.412)
**Refinement**		
Reflections	24908 (2477)	32099 (3179)
Reflections (R-free)	1206 (133)	1336 (135)
R-work (%)	17.70 (33.68)	19.30 (39.02)
R-free (%)	21.00 (33.18)	22.20 (43.25)
No. non-hydrogen atom	3202	3130
macromolecules	2884	2909
ligands	37	14
solvent	281	207
Protein residues	345	347
r.m.s.d		
Bond lengths (Å)	0.005	0.004
Bond angles (°)	1.06	0.98
Ramachandran plot		
Favored (%)	98.22	98.82
Allowed (%)	1.78	1.18
Outliers (%)	0.00	0.00
Average B-factor	30.64	29.13
Macromolecules	30.03	28.55
Ligands	47.57	52.44
Solvent	34.73	35.64

Statistics for the highest-resolution shell are shown in parentheses.

### Active site conformations

Loops 1 and 7 of A3B(187–378)QM_Δloop3 in the new crystal form show different conformations from those observed previously^15^. In our previous structures, the side chains of Arg211 from loop 1 and Tyr315 from loop 7 project into the ssDNA-binding groove and are stacked on each other, leaving a very small portal to the active site pocket (Figs 1A and 2A). In both A and B chains of the new structures, the loop 1 residue Arg211 is flipped out and, instead, the adjacent residue Arg212 projects into the DNA-binding groove and interacts with loop 7 Tyr315 (Figs 1C,D and 2C,D). In chain A, the positioning of Tyr315 side chain is essentially as observed in previous structures (*e.g*., PDB ID 5CQI), pointed towards loop 1 (χ_1_ torsion angle of Tyr315 ≈ −60°, *gauche* ^+^) and blocking the –1 thymine (T) base-binding pocket (Figs 1C, 2C and Supplementary Figs S1A,C, S2C and S3A). In contrast, Tyr315 in chain B shows an alternative conformation differing by a rotation about the Cα-Cβ bond, in which the Tyr315 side chain is flipped away from loop 1 (χ_1_ ≈ 180°, *trans*) (Figs 1D, 2D and Supplementary Figs. S1B,D, S2D, S3B and S4). Importantly, this *trans* conformation of Tyr315 was also observed in the open state crystal structure of the A3Bctd active site bound to a TC-containing ssDNA substrate (Figs 1B, 2B and Supplementary Fig. S2B), where the Tyr315 side chain lines a hydrophobic pocket that accommodates the –1 T base^25^. However, in our new apo A3Bctd crystal form, Tyr315 in this alternative conformation maintains the cation-π stacking interaction with the guanidinium group of Arg212. Accordingly, Arg212 in B chain is positioned differently from that in A chain, but is still blocking the ssDNA-binding groove. Of note, the two datasets we collected (Table 1) using different cryo-protectants showed a slight difference in the conformation of Tyr315 in the B chain. In the structure obtained with ethylene glycol as the cryo-protectant (5SXH), electron density suggested co-existence of both the *trans* and *gauche* conformers, and we were able to refine the structure with multiple conformations for Tyr315 (Supplementary Fig. S1D). On the other hand, the second dataset (5SXG) shows predominantly the *trans* conformer, likely due to a 1,3-propanediol molecule (cryo-protectant) bound within the ssDNA –1 T pocket and shifting the conformational equilibrium (Supplementary Figs. S1B and S3B).

In addition to Arg211 and Arg212, another pair of arginines, Arg210 from loop 1 and Arg311 from loop 7, switch positions between the crystal forms. In the original structures (*e.g*., PDB ID 5CQI), the Arg210 side chain is positioned close to the C-terminus of α1, tucked behind Trp281 that makes key hydrophobic contacts with the –1 T and target (0) C bases of the ssDNA substrate^25^. Arg311 across the ssDNA-binding groove is hydrogen-bonded to the carbonyl group of Val208 from loop 1 (Fig. 2A and Supplementary Fig. S2A), a part of the ^206^PLV^208^ stretch unique to A3B (alignment in Fig. 3). In the new crystal form, Arg210 is released and flipped out, replaced by Arg311 that in turn occupies the pocket between α1 and Trp281. The latter conformation of Arg311 is also observed in the crystal structure of the ssDNA-bound A3B variant, as well as for the corresponding residue Arg128 in A3A-ssDNA complex, suggesting that it represents the ‘open’ active state. The anchoring of Arg210 behind Trp281 and the Arg311-Val208 hydrogen-bond contribute to stabilizing the closed conformation of the loops 1 and 7 incompatible with binding ssDNA, along with the interaction between Arg211/212 and Tyr315. The three distinct conformations of the loops 1 and 7 observed in the apo (DNA-free) A3B(187–378)QM_Δloop3 structures collectively represent possible modes of regulated ssDNA access to the A3B active site, in which the closed state of either loop 1 or 7 (or both together) is incompatible with substrate binding.

Another loop 7 residue beside Tyr315 with variation in side chain orientation is Asp314, which defines the TC sequence preference of A3B by hydrogen bonding to the –1 T base^25^. In the A chain of the new crystal form of A3B(187–378)QM_Δloop3, the Asp314 side chain is pointed directly towards Arg372 from α6, with its carboxyl group rotated away from where the thymine base would bind (Figs 1C and 2C). In the B chains, the Asp314 side chain is positioned as in the DNA-bound structure or the previously reported apo structures in the closed conformation (*e.g*., PDB ID 5CQI), with only one of the carboxyl oxygen atoms involved in hydrogen-bonding with Arg372 and leaving the other for hydrogen-bonding with the –1 T (Figs 1D and 2D). Thus, like Tyr315, Asp314 appears to be capable of sampling two conformations and only one is compatible with ssDNA binding (Supplementary Fig. S2). However, to test this idea we made an A3Bctd variant with R372A and, in comparison to the parental construct with an intact Arg372, the Ala372 derivative showed no detectable change in enzymatic activity suggesting that the latter conformation may be preferred in solution (Supplementary Fig. S5A). MD simulations (detailed below) also show that the Asp314 side chain is predominantly (>92% of the time) in the gauche^−^ conformation compatible with hydrogen-bonding with the –1 T base (χ_1_ angle ≈+ 60°; Supplementary Fig. S5B). These data combine to indicate that the Asp314-Arg372 interaction is unlikely to have a significant role in regulating A3B activity.

### Active site dynamics

Consistent with the multiple conformations observed in our A3Bctd crystal structures, recent NMR studies have indicated that the loop 1 region, including the Arg210-Arg212 stretch, can be highly mobile^33^. To further investigate the dynamics of the loops surrounding the active site, we performed all-atom explicitly solvated MD simulations of wild-type A3Bctd. An analysis of the fluctuation of the positions of residues about their mean position (root mean squared fluctuation [RMSF]) over three independent 1 μs MD trajectories shows three highly flexible regions in A3Bctd (Fig. 4B): the loop 1 region spanning residues Asn204 to Gln213, the loop following β1 and the first half of the interrupted β2 (including Asp224 to Met231 which are disordered in the new crystal form), and the loop 3 region truncated in our crystal structures but shown to be highly flexible in NMR studies of A3Bctd and A3A^33–35^. Wild-type A3A MD simulations also showed high mobility of the latter two regions, however, A3A loop 1 had limited flexibility compared to the corresponding loop 1 region of A3Bctd (Fig. 4C).

**Figure 5:**
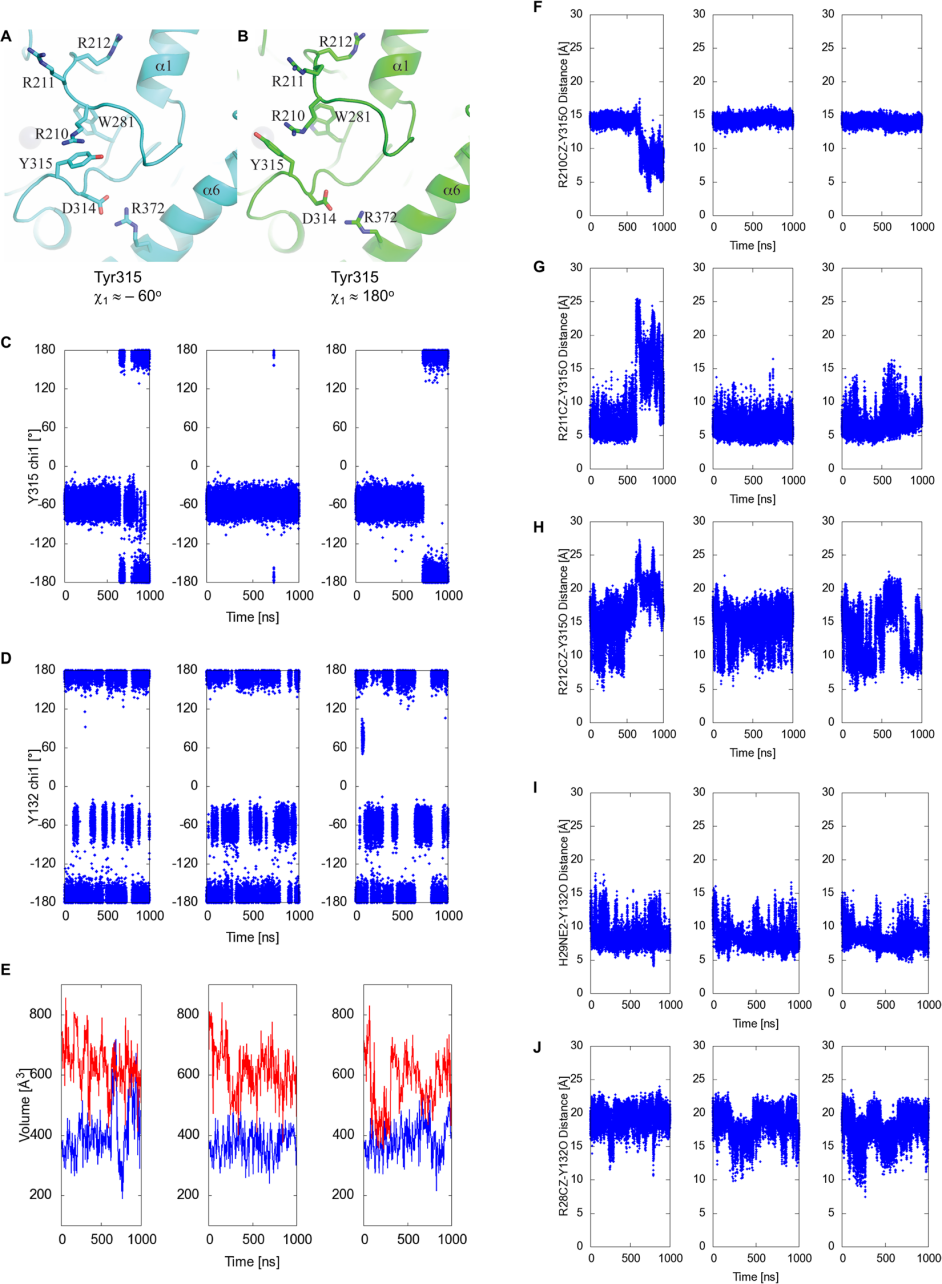
A3Bctd and A3A active site dynamics studied by MD simulations. (**A**,**B**) Novel closed A3B active site conformations observed in MD simulations. Arg210 interacts with Tyr315 in either the closed *gauche* ^+^ (**A**) or open *trans* (**B**) conformation. (**C**,**D**,**E**) Transitions of A3Bctd Tyr315 (**C**) or A3A Tyr132 (**D**) χ_1_ angle and active site volumes (**E**) in the apo A3Bctd (depicted in blue) and A3A (red) MD simulations. His29 of A3A is fully protonated (HIP29). Note that Tyr315 conformational change to trans (χ_1_ angle ≈ 180°) results in a more open A3Bctd active site with larger volume values. The trajectories for 3 independent 1-μs simulations are plotted separately. (**F**,**G**,**H**) Distance between each of the arginine residues from the R210-R211-R212 triad (CZ atom) and the Tyr315 backbone O atom in apo A3Bctd MD simulations. (**I**) Distance between His29 (NE2 atom) and the Tyr132 backbone O atom in apo A3A MD simulations with His29 fully protonated (HIP29). (**J**) Distance between Arg28 (CZ atom) and the Tyr132 backbone O atom in apo A3A MD simulations with His29 fully protonated (HIP29).

The starting model for restoring wild-type A3B and performing MD simulations was a tightly closed structure (PDB ID 5CQI) (Methods). Throughout most of the simulations, Arg211 maintains an association with Tyr315 as indicated by the Arg211 NZ – Tyr315 O (backbone carbonyl oxygen) distance below 10 Å (Fig. 5G). However, novel closed active site conformations were observed in the simulations. Between 700 and 1000 ns in the trajectory #1 shown in Fig. 5C,F–H, the Arg211 side chain flips away from loop 7 and Arg210 instead projects into the DNA-binding groove and interacts with Tyr315 in either *gauche* ^+^ or *trans* conformation (Fig. 5A,B). The association of Arg210 and Tyr315 is accompanied by a rearrangement of the active site pocket, which includes rotation of the Trp281 side chain. Although this conformation was not captured in any of the A3Bctd crystal structures, the side chain of the corresponding residue Trp94 in an APOBEC3G ntd crystal structure shows a similar rotated conformation^36^. These observations demonstrate plasticity of the APOBEC active site and suggest that Arg210 could also be part of the ‘Arg switch’ of A3B involving Arg211 and Arg212 (Fig. 5F–H).

As described above, the ‘open’ *trans* conformation of Tyr315 from loop 7 is a prerequisite for A3Bctd to bind ssDNA substrates. A3A and A3Bctd share identical loop 7 sequences (Fig. 3), and a comparison between the crystal structures of apo and ssDNA-bound A3A also shows reorientation of Tyr132 from loop 7, analogous to the closed *vs*. open transitions of A3B Tyr315^25,30,37^. The MD simulations show that the apo form of A3Bctd Tyr315 side chain mostly maintains the ‘closed’ *gauche* ^+^ conformation (~84% of the time) with occasional and transient switching to the *trans* conformation (Fig. 5C, and Supplementary Video 1). Interestingly, MD simulations of apo A3A show Tyr132 to be predominantly in the open *trans* conformation (~66% and ~42% for protonated and deprotonated His29 MD simulations, respectively) with more frequent interconversion between the two states likely reflecting a lower activation barrier (Fig. 5D, Supplementary Fig. S6A, and Supplementary Video 2). The critical ssDNA-binding residue of A3A, His29, also showed a dynamic behavior with intermittent interaction with the Tyr132 side chain (Fig. 5I, Supplementary Video 2), whereas Arg28, corresponding to A3B Arg211, is consistently flipped away from the DNA-binding groove (Fig. 5J).

Overall, the MD trajectories indicate that loop 1 of A3Bctd is highly dynamic and can sample multiple conformations, including those observed crystallographically as well as other closed and open states. In contrast, the shorter loop 1 of A3A appears to be more rigid, which leaves the A3A active site in a more open conformation. The flexibility of A3Bctd loop 1 and its various interactions with Y315 results in smaller active site volumes (MD simulation average of 389 ± 74 Å^3^) (Fig. 5E). A3A, on the other hand, samples significantly larger active site volumes, and only briefly samples closed active site states (averages of 607 ± 90 Å^3^ and 640 ± 75 Å^3^ for His29 protonated and deprotonated MD simulations, respectively) (Fig. 5E, Supplementary Fig. S6A). A3A has higher catalytic activity at lower pH reaction conditions^38^, as reported originally for A3Gctd with an analogous histidine in loop 1^39^. Thus, a fully protonated His29 side chain in lower pH conditions may enhance A3A activity by not only interacting more optimally with ssDNA but also by facilitating opening of the active site pocket (*e.g*., by tilting the Y132 conformational equilibrium to favor the open state). Of note, MD simulations indicated that the highly flexible loop 3 region does not make specific interactions with loop 1 or loop 7 residues, and therefore has no effect on their conformation or dynamics in either A3B or A3A.

### A3B derivatives with engineered loop 1

The highly homologous A3A and A3Bctd differ in loop 1 amino acid sequences (alignment in Fig. 3). A3B loop 1 features a stretch of hydrophobic residues (^206^PLVL^209^) followed by a hydrophilic, arginine-rich stretch (^210^RRRQ^213^). The loop 1 of A3A is shorter by 3 amino acids and residues important for ssDNA-binding, including His29, are not present in A3Bctd. The residues from the shorter loop 1 of wild-type A3A do not block the DNA-binding groove, rendering its active site more open in the absence of ssDNA compared to the mostly closed A3Bctd active site (Fig. 5E). Given these structural observations, we reasoned that the higher ssDNA deaminase activity of A3A may not only be because of having a more optimal set of ssDNA-binding residues (His29 in particular), but also be due to higher accessibility of the active site, as exemplified by conformations of A3A Tyr132 versus A3B Tyr315 as observed in MD simulations (Fig. 5C,D).

**Figure 6:**
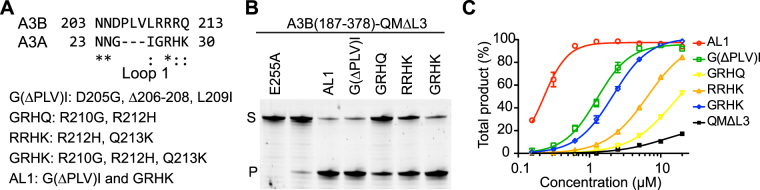
ssDNA deamination activities of A3Bctd loop 1 variants. (**A**) Loop 1 amino acid sequences for A3Bctd and A3A, and a nomenclature key for the analyzed constructs. (**B**) Gel image of single time point DNA cytosine deamination activity data for the indicated constructs. E255A is catalytically inactive (negative control). S; substrate, P; product. (**C**) A representative dose response experiment showing increased accumulation of ssDNA deamination products with increasing enzyme concentrations for the indicated constructs (n = 3 parallel experiments with mean ± SD shown; error bars not shown are smaller than the symbols used to plot the means).

To further probe the possible regulatory roles of observed loop 1 conformations of A3Bctd on ssDNA deaminase activity, we examined the activities of a series of A3Bctd constructs, with various segments of loop 1 substituted from A3A. These substitutions were made in the background of A3B(187–378)QM_Δloop3, which has lower activity than wild-type A3B (full-length) or A3Bctd^15^. The C-terminal half of A3B loop 1 including the arginine stretch, Arg210/Arg211/Arg212/Gln213 corresponds to Gly27/Arg28/His29/Lys30 of A3A (Fig. 3). Changing the two non-conserved arginines Arg210/Arg212 to Gly/His (GRHQ) made A3B(187–378)QM_Δloop3 slightly more active, selectively in a lower pH condition (Fig. 6). Changing Arg212/Gln213 to His/Lys (RRHK) had a similar, but more pronounced effect. Combining all 3 substitutions (GRHK) had additive effects as expected, which made A3B(187–378)QM_Δloop3 more active in both pH conditions. His29 of A3A is a critical ssDNA-binding residue with dual backbone phosphate contacts^25,30^. Therefore the higher activities and clear pH-dependence of the chimeric proteins likely reflect, at least in part, the favorable DNA contacts made by the introduced His residue, and possibly loss of the interactions between Arg210 or Arg212 and Tyr315 that maintain a closed active site conformation. These observations are also consistent with the reported importance of Q213K substitution in converting A3B to a more active form^17,31^ and the observation that an H29R substitution lowers A3A activity^33^.

In the A3A-ssDNA complex structure, Lys30 is at the C-terminal end of loop1 and not involved in a direct DNA contact. Thus, the effect of the Q213K substitution in enhancing A3B activity could be due to altered loop 1 conformation or dynamics. All apo A3B(187–378)QM_Δloop3 structures determined to date show dual hydrogen-bonds between Gln213 side chain and Asn240 backbone atoms (Fig. 2A,C,D). This Gln213 positioning precludes the active conformation of Asn240, which makes an interaction with the backbone of a ssDNA substrate (Fig. 2B), and may explain the effect the Q213K substitution. The inter-residue distance plots (Supplementary Fig. S7) show proximities of A3B Gln213 to Asn240 and A3A Lys30 to Asn57 in the MD simulations. However, analysis of side chain geometries with the program visual molecular dynamics (VMD)^40^ showed that Gln213 in A3B maintains at least one hydrogen-bond to other residues in 21% of the trajectory (with the most persistent hydrogen-bonds to Asn240, Gln241, Tyr215 and Asn201), while the corresponding Lys30 in A3A forms hydrogen-bonds in only 1% of the trajectory and no salt-bridge interactions.

Interestingly, we also observed a strong enhancement of A3B(187–378)QM_Δloop3 ssDNA deaminase activity by truncating the N-terminal half of loop 1, without changing the putative DNA-contacting residues including the arginine-rich stretch. The G(ΔPLV)I construct, which lacks the 3 extraneous amino acids present in the upstream portion of loop 1, is in fact the second most active construct in the *in vitro* deamination assay, next to a construct in which the entire loop 1 from A3A is swapped into A3Bctd (AL1 in Fig. 6). Notably, the truncated residues include Val208, whose backbone carbonyl group is hydrogen-bonded to Arg311 to maintain the closed active site in the original (5CQI) A3B(187–378)QM_Δloop3 crystal structure^15^ (Figs 1A and 2A, Supplementary Fig. S2A). The hydrogen-bond analysis of MD trajectories also revealed dynamic behavior the ^206^PLV^208^ region of A3B with three persistent inter-residue hydrogen-bonds; between Leu207 backbone and Asp205 side chain (in 26% of the trajectory), Val208 backbone and Arg311 side chain (in 14%), and Val208 backbone and Asp205 backbone (3%). These results strongly suggest that the higher activity conferred by the A3A loop 1 is not solely dependent on the identities of the amino acids in direct contacts with DNA, but also likely depends on factors that affect the positioning and accessibility of these residues.

## Discussion

Through our previous and present studies, we have shown that the loops 1 and 7 of A3Bctd can adopt several distinct ‘closed’ conformations, in which the DNA-binding groove is blocked. The binding of A3Bctd to relevant ssDNA substrates requires multiple conformation changes. First, Asp314 and Tyr315 adopt *gauche* ^***−***^ (χ^1^ ~ 60°) and *trans* (χ^1^ ~ 180°) conformations, respectively, to form the pocket to accommodate –1 T. Second, the hydrogen bond between Arg311 and Val208 is broken. Third, Gln213 moves away to allow conformational change of Asn240 for interacting with DNA backbone. Fourth, Arg210 tucked behind Trp281 is replaced by Arg311 and released. Finally, Arg210/Arg211/212 side chains become unstacked from Tyr315. In our MD simulations of apo A3Bctd, we did not observe such fully ‘open’ conformations of the active site that simultaneously satisfy all conditions listed above. However, snapshots of an open active site, where none of the Arg210–211–212 triad interacts with Tyr315 side chain and Tyr315 is in *trans* conformation, were obtained at transitions between distinct closed states (Supplementary Video 1). Although understanding the precise mode of ssDNA-binding by the natural A3B loop 1 will require further investigation, one or more of these arginine residues is likely to interact with ssDNA analogously to A3A His29 (*i.e*., clamp-like positioning to hold ssDNA in place). Thus the closed conformations may also serve to sequester critical DNA-binding residues. Conversely, it is possible that an opening of the A3Bctd active site is induced by the negative electrostatic potential of the backbone of an approaching ssDNA substrate interacting with the highly positively charged arginine stretch. In addition, the critical interaction made by the –1 T base has the potential to stabilize the *trans* conformation of Tyr315 and lock it in an open conformation.

Our biochemical analyses show that the attenuated ssDNA deaminase activity of A3Bctd, as compared to that of A3A, is due to the compounded effects of different amino acids in the C-terminal half of loop 1 and the additional residues in the N-terminal half of the longer A3Bctd loop 1. Our structural and MD simulation data further suggest that the extraneous residues provide A3Bctd loop 1 with the flexibility to sample multiple closed active site states. Interestingly, a sequence alignment of A3Bctd shows that R210 and R212 of human A3Bctd are not conserved, and many primates in fact have Gly and His, respectively, at these amino acid positions to more closely resemble A3A (Supplementary Fig. S8). However, the overall length of loop 1 and amino acid residues Val208, Arg211, and Gln213 are conserved amongst primate A3B enzymes, likely reflecting a regulatory role for A3Bctd loop 1. We hypothesize that the ssDNA-binding affinity and deaminase activity of A3B has evolved to limit the pool of molecules in the active state in order to balance beneficial physiological functions in innate immunity with potentially detrimental mutagenic effects in the nucleus.

Given the dominant role of A3B-mediated genomic mutations in cancer evolution, activity inhibition is an attractive strategy to slow down tumor evolution. As our results suggest that the conformational equilibrium of the apo (non-DNA bound) form of A3Bctd is skewed toward being closed, development of a high-affinity competitive inhibitor that targets the active site could be challenging. On the other hand, we envision that any of the fully or partially closed inactive states could be stabilized by a small molecule inhibitor, which would further shift the equilibrium toward one of the inactive conformations to inhibit enzyme activity. Such allosteric mechanisms of inhibition may be advantageous in achieving high selectivity against A3B among highly related APOBEC3 family members in human cells.

## Methods

### Crystallization and structure determination

A3B(187–378)QM_Δloop3 was expressed and purified as reported^15^, except the protein used in the present study was expressed with an N-terminal 6xHis-SUMO tag. The tag was removed by Ulp1 treatment during purification, generating a protein with no vector-derived amino acids. Thin needle-cluster crystals were obtained by the hanging drop vapor diffusion method, using reservoir solution containing 0.1 M malonate-imidazole-borate buffer (pH 4 to 5) and 25% PEG 1,500. The crystals were flash-cooled for data collection in liquid nitrogen using ethylene glycol or 1,3-propanediol as a cryoprotectant. X-ray diffraction data were collected at the Advanced Photon Source NE-CAT beamlines 24-ID-C/E and processed using XDS^41^ or HKL2000^42^. Molecular replacement calculation with PHASER^43^ using our previous A3B(187–378)QM_Δloop3 structure (PDB ID: 5CQI) as the search model identified two protein molecules in the asymmetric unit. The atomic models were built using COOT^44^ and refined using PHENIX^45^. A summary of data collection and model refinement statistics is provided in Table 1. In the new crystal form (PDB ID: 5SXH/5SXG), loop 1 residues make less extensive lattice contacts than in the original crystal form (5CQI)^15^. However, in both cases the flipped-out arginine residue (Arg211 or Arg212) is stabilized by a hydrogen-bond with a neighboring molecule. These lattice contacts surrounding the active site in both crystal forms are shown in Supplementary Fig. S9.

### Molecular Dynamics Simulations

The wild-type A3Bctd system was generated based on the apo A3B(187–378)QM_Δloop3 crystal structure, PDB ID: 5CQI^15^. Amino acid substitutions introduced for protein solubility and crystallization were reverted *in silico* and the missing loop 3 residues were built using homology modeling in the Schrodinger suite (Small-Molecule Drug Discovery Suite 2017-1, Schrödinger, LLC, New York, NY, 2017). All A3A systems were generated based on the DNA-bound A3A crystal structure (PDB ID: 5SWW^25^). Ala72 was computationally reverted to a protonated catalytic Glu to generate the wild-type A3A. The missing residues 1 to 10 were added using the lowest energy model of apo A3A NMR structure as a template (PDB ID: 2M65^34^). For A3A, two systems were generated. The first has DNA deleted from the active site and His29 doubly-protonated (called HIP29). The second has DNA deleted from the active site and His29 singly-protonated (called HID29).

Protonation states of all titratable amino acids were determined based on PropKa analysis in VMD^46^. All crystallographic water molecules were retained unless a steric clash with protein was observed. The amino acids coordinating zinc were positioned using the cationic dummy atom model^47^. Each protein was solvated in a TIP3P^48^ water box with 10 Å buffer and Amber FF14SB force field^49^ was used to build the systems. The final systems consisted of 10,693 atoms for A3B apo system and 10,733 atoms for A3A apo system. All simulations were performed with Amber14 suite (D.A. Case, V. Babin, J.T. Berryman, R.M. Betz, Q. Cai, D.S. Cerutti, T.E. Cheatham, III, T.A. Darden, R.E. Duke, H. Gohlke, A.W. Goetz, S. Gusarov, N. Homeyer, P. Janowski, J. Kaus, I. Kolossváry, A. Kovalenko, T.S. Lee, S. LeGrand, T. Luchko, R. Luo, B. Madej, K.M. Merz, F. Paesani, D.R. Roe, A. Roitberg, C. Sagui, R. Salomon-Ferrer, G. Seabra, C.L. Simmerling, W. Smith, J. Swails, R.C. Walker, J. Wang, R.M. Wolf, X. Wu and P.A. Kollman, 2014, AMBER 14, University of California, San Francisco).

Each system was first relaxed by 41,500 steps of minimization followed by 4 consecutive restrained molecular dynamics simulations. In the first 500 steps of minimization, only hydrogen atoms were minimized leaving all other atoms fixed. In the second 500 steps, all hydrogen atoms, water molecules and ions (except Zn ion) were minimized. In the third 500 steps, all atoms except the protein backbone were minimized. The entire systems were then minimized without any restraints for 40,000 steps. After the minimizations, each system was slowly heated up from 0 K to 310 K using a positional restraint of 3.0 kcal/(mol. Å^2^) on all non-hydrogen atoms of protein except the zinc-coordinating residues. Each system was then equilibrated in three consecutive restrained MD simulations at 310 K and 1 atm using restraints of 3.0, 2.0 and 1.0 kcal/(mol. Å^2^) on all non-hydrogen atoms of protein except the zinc-coordinating residues. After this multi-step system relaxation protocol, production MD simulations were performed without any restraints for 1-μs with 2 femtosecond time steps. Temperature was maintained at 310 K using Langevin dynamics with a collision frequency of 5 ps^**−**1^ and pressure was maintained at 1 atm using isotropic position scaling with a pressure relaxation time of 2 ps. Long-range electrostatics was treated by the Particle Mesh Ewald method^50^ and a non-bonded cutoff of 10 Å was used. The SHAKE algorithm was used to constrain the bonds involving hydrogen atoms^51^.

Using the same protocol detailed above, three independent copies of 1-μs MD simulations for each of apo A3Bctd, apo A3A with HIP29 and apo A3A with HID29 were performed with different random number seeds. RMSD of C-alpha atoms with respect to the initial frame for each system is plotted to show the stability of MD simulations (Supplementary Fig. S6B). For each system, the total MD trajectory consisted of 60,000 frames recorded with 0.05 ns intervals. RMSD, RMSF, distance and dihedral angle analysis were performed using cpptraj software^52^. The POcket Volume MEasurer 3.0 (POVME) program was used to calculate the volumes of the ssDNA binding pocket^53–55^. The pocket was defined by a set of 5 overlapping inclusion spheres with radii from 5 to 7 Å that cover the volume traversed by bound ssDNA. POVME was run with all default settings, no enforced seed/contiguous regions, a voxel grid spacing of 1.0 Å, and the ConvexHullExclusion option set to “none”. Frames were taken at 5 ns intervals from the simulations for pocket shape analysis, resulting in 600 snapshots from each simulation being analyzed. All snapshots from the protein simulation were aligned to a single reference structure by their alpha carbons to allow for comparison between A3A and A3B. As the simulated binding pockets are large and flexible, the absolute value of the pocket volume may not agree with volume measurements taken by other programs. However, the trajectory alignment and use of identical inclusion regions enabled accurate comparison of POVME volumes between the simulations of A3 proteins.

### DNA deaminase assays

A3B(187–378)QM_Δloop3 with various loop 1 modifications were expressed with a C-terminal 6xHis-tag (LEHHHHHH) using the pET24a vector and purified as reported^15^. A3B(187–378)L230K/F308K and A3B(187–378)L230K/F308K/R372A were expressed with the N-terminal 6xHis-SUMO tag and purified using nickel-affinity and gel-filtration chromatography. The 6xHis-SUMO tag was not removed for activity assays. Activity assays were performed in 10 mM citrate buffer pH 5.5, 100 mM NaCl, 1 mM EDTA, 100 μg/mL BSA with 800 nM substrate ssDNA (5’-ATTATTATTATTCAAATGGATTTATTTATTTATTTATTTATTT-fluorescein). Incubations proceeded for 1 hour at 37 °C, followed by heating to 95 °C for 10 minutes. pH was normalized and uracils removed by adding Tris-Cl pH 8.0 to a final concentration of 30 mM and 0.5 U/rxn of uracil-DNA glycosylase (NEB). Uracil excision reactions were performed for 20 minutes at 37 °C. DNA was cleaved by the addition of sodium hydroxide to 100 mM and heating for 10 minutes at 95 °C. Reaction products were separated by 15% TBE-Urea PAGE and gels were scanned on a Typhoon FLA-7000. Band intensities were quantified using ImageQuant TL (GE LifeSciences).

### Data availability

The atomic coordinates and structure factors for the crystal structures reported in this paper have been deposited in the Protein Data Bank (PDB) under accession codes 5SXH and 5SXG. Uncropped gel images for Fig. 6B and Supplementary Fig. S5A are available in the online version of the paper. Other data will be available upon request.

## Electronic supplementary material

Supplementary Figs

Supplementary Video 1

Supplementary Video 2

Uncropped gel images
