# Supplementary material for: Conformational Switch Regulates the DNA Cytosine Deaminase Activity of Human APOBEC3B
Source: Sci Rep. 2017 Dec 12;7:17415. doi: 10.1038/s41598-017-17694-3 (PMC5727031; doi:10.1038/s41598-017-17694-3)
Supplement: Supplementary file 1 — Supplementary Figs [file 41598_2017_17694_MOESM1_ESM.pdf]

Supplementary Information

Conformational Switch Regulates the DNA Cytosine Deaminase Activity of Human APOBEC3B

Shi, Demir, Carpenter et al.

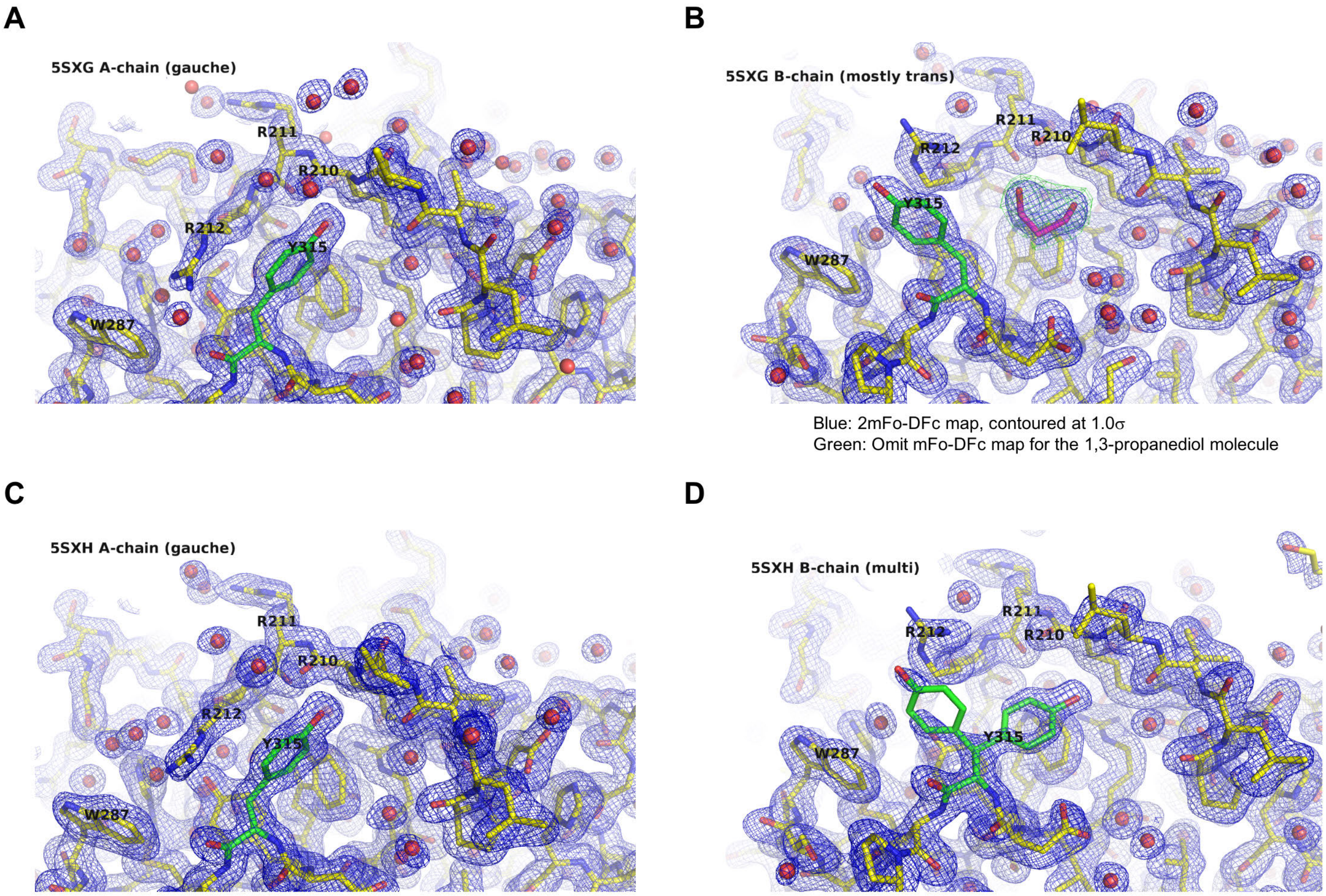

Fig. S1: Electron density maps for the new crystal structures of A3B(187-378)QM_Δloop3. The σA-weighted 2mFo-DFc electron density map contoured at 1.0 σ is shown in blue mesh. In (C), mFo-DFc omit map at 3.0 σ for the 1,3-propanediol molecule is overlaid in green. The red spheres represent water molecules.

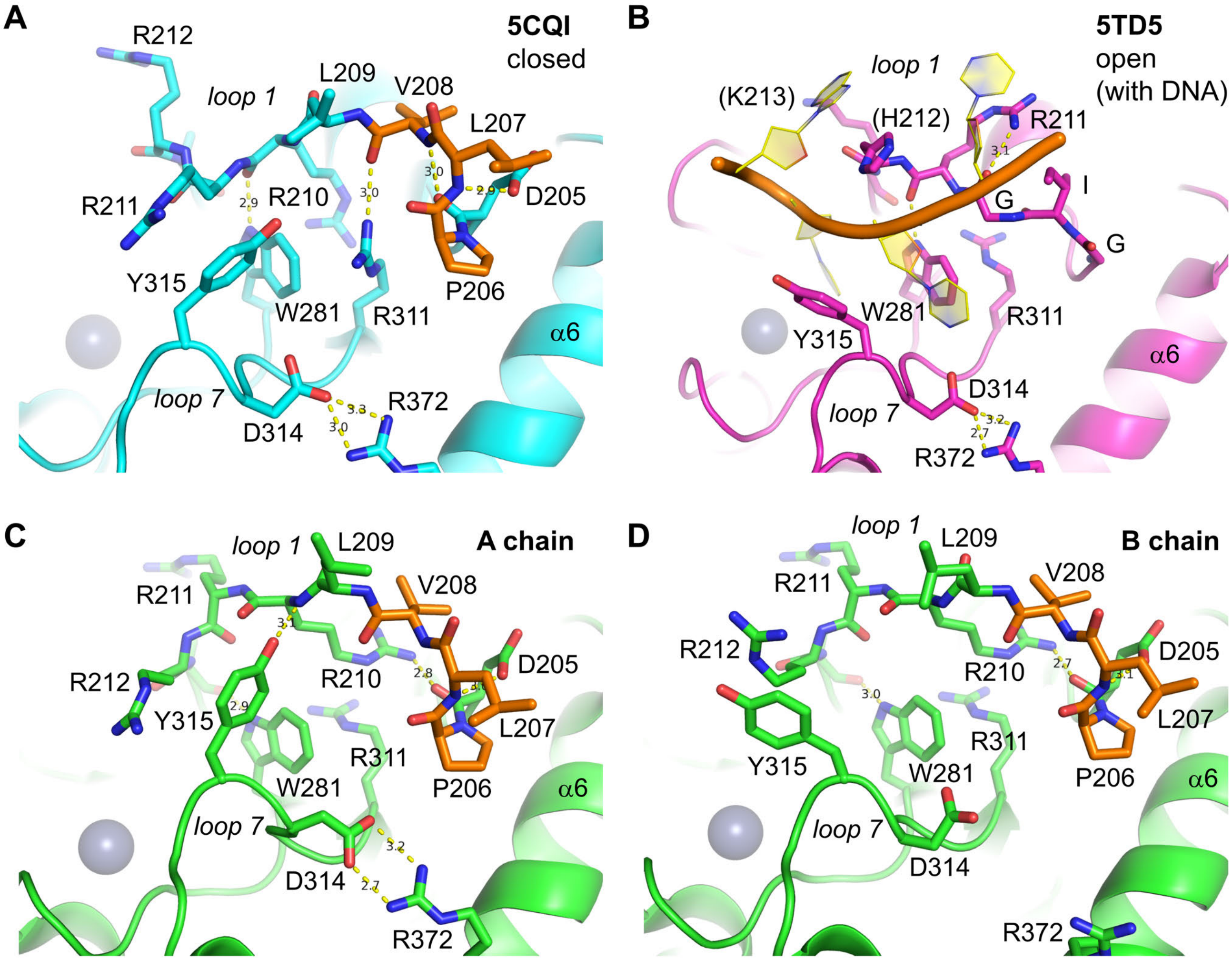

Fig. S2: Additional views of the closed A3Bctd active site (A, C, D) and that in complex with ssDNA (B). The panels A-D correspond to those in **Fig. 2** and show all residues from loop 1 and their interactions. The ^{206}PLV^{208} stretch is highlighted in orange. Note two alternative modes of closed A3Bctd active site (A *vs*. C/D) featuring an "Arg switch (Arg311/Arg210)" and the Val208-Arg311 hydrogen-bond stabilizing the tightly closed active site in (A). The shorter loop 1 in the ssDNA-bound structure (B) is from A3A.

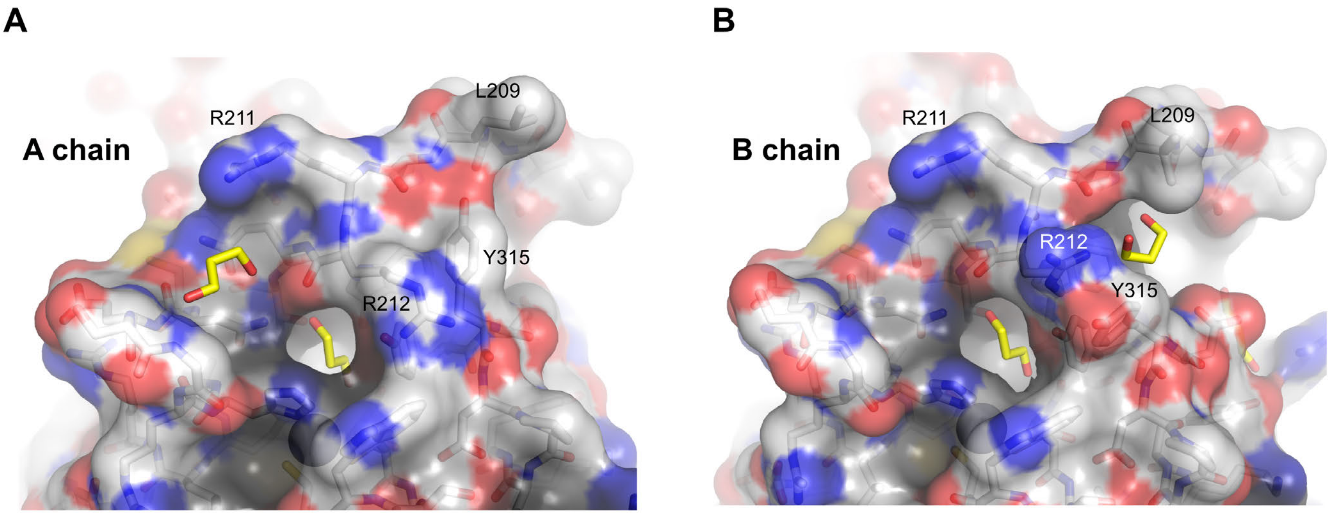

Fig. S3: Surface representation of the closed conformations of A3Bctd in the new crystal form (5SXG). In both cases, R212 interacts closely with Y315. Shown in the yellow sticks are 1,3-propanediol molecules.

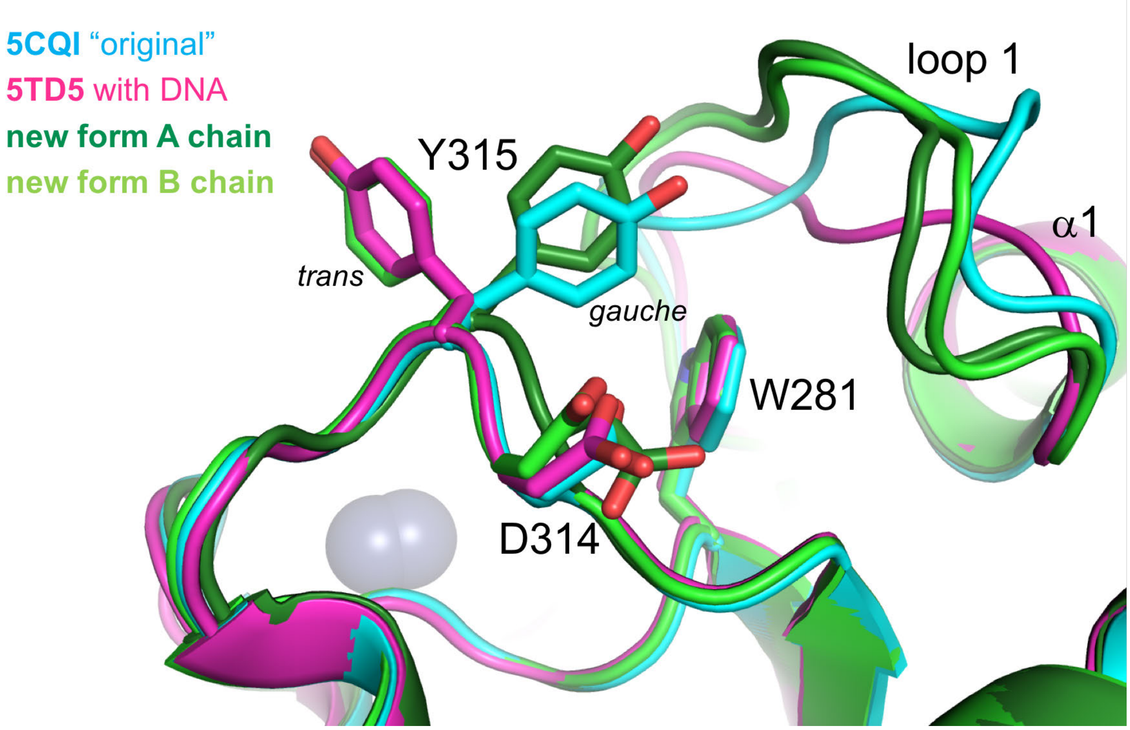

Fig. S4: The alternative (*trans* vs. *gauche*+) Tyr315 conformations in various crystal structures. Asp314 and Trp281 side chains are also shown in sticks. The gray spheres represent the zinc ion in the active site.

A

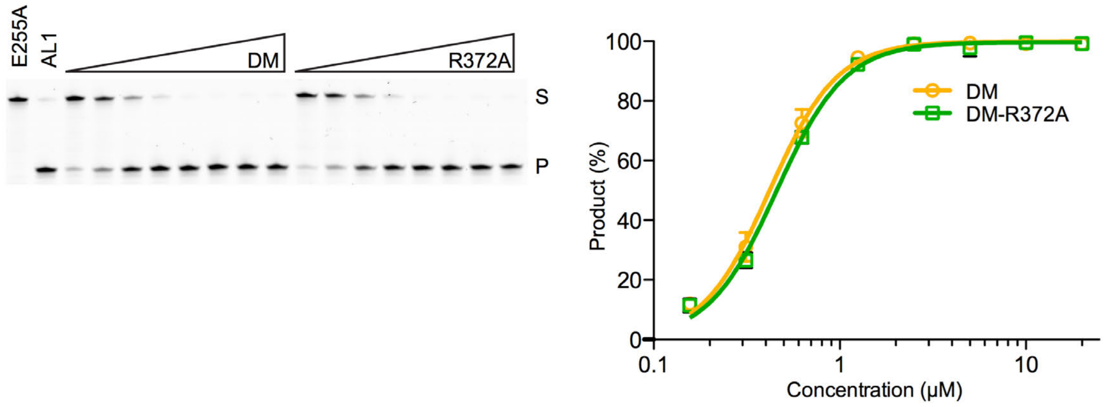
E255A
AL1
DM
R372A
S
P
100
80
60
40
20
0
Product (%)
0.1
1
10
Concentration (μM)
DM
DM-R372A

B

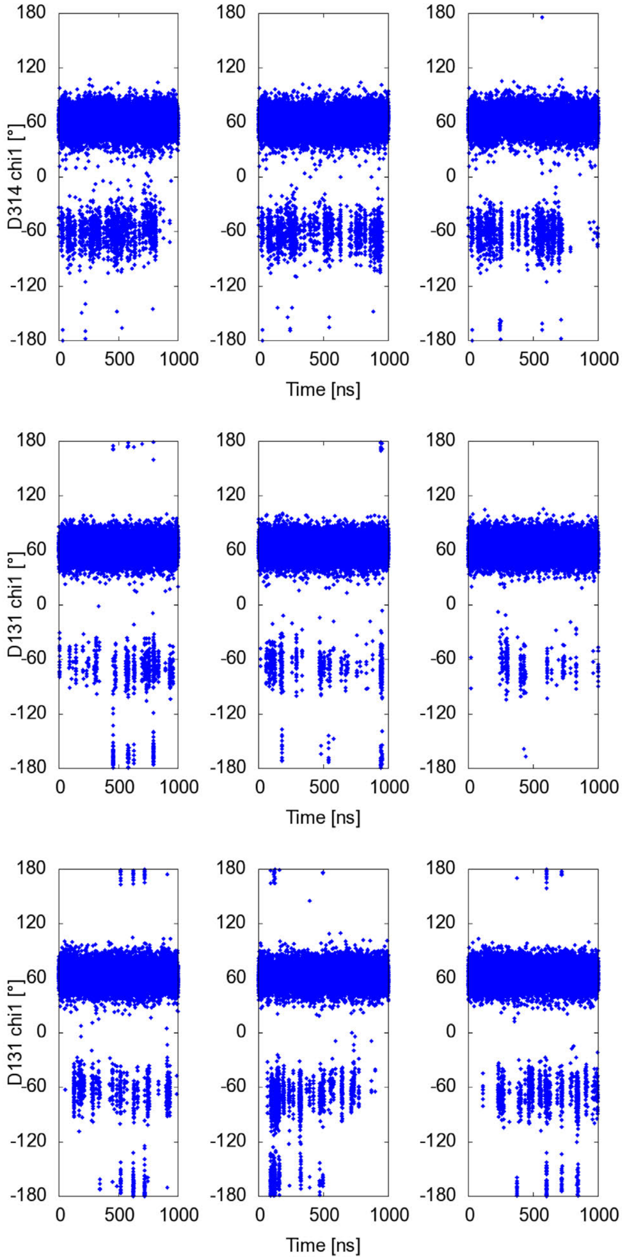
180
120
60
0
-60
-120
-180
D314 chi1 [°]
D131 chi1 [°]
D131 chi1 [°]
0
500
1000
Time [ns]

Fig. S5: A3B D314-R372 interaction does not have a regulatory role.

(A) R372A amino acid substitution has no significant effect on A3Bctd activity. A comparison of the in *vitro* ssDNA deaminase activities of sumo-A3Bctd-DM (L230K/F308K) and sumo-A3Bctd-DM+R372A. R372 interacts with the key ssDNA-binding residue D314 involved in the -1 T recognition.

(B) D314 is predominantly in the conformation compatible with ssDNA-binding. Asp314 (Asp131 in A3A) χ_1 angle in MD simulations of A3Bctd, A3A_apo_HIP29 and A3A_apo_HID29, respectively, from top to bottom. The χ_1 angle of ~60° (gauche$^-$) corresponds to the 'open' conformation compatible with ssDNA-binding.

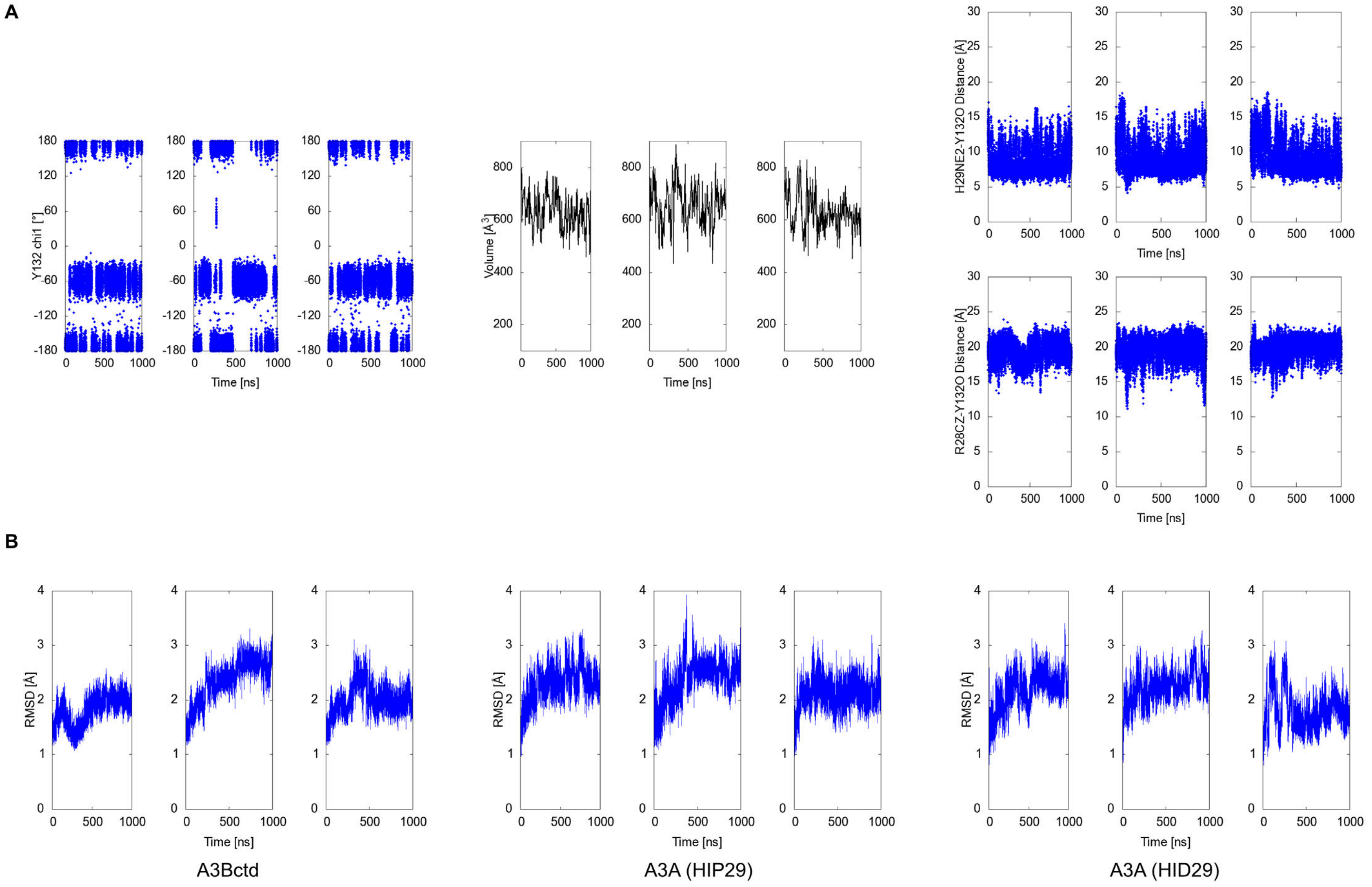

Fig. S6: Additional MD simulations data.

(A) Tyr132 χ_1 angle, active site volume, and the distance between His29 or Arg28 side chain and Tyr132 backbone (corresponding to **Fig. 5D**, **E**, **I**, and **J**) in apo A3A HID29 MD simulations.

(B) RMSD of all Cα atoms with respect to the initial frame showing stability of the MD simulations. For each system, three independent microsecond-long simulations are plotted separately.

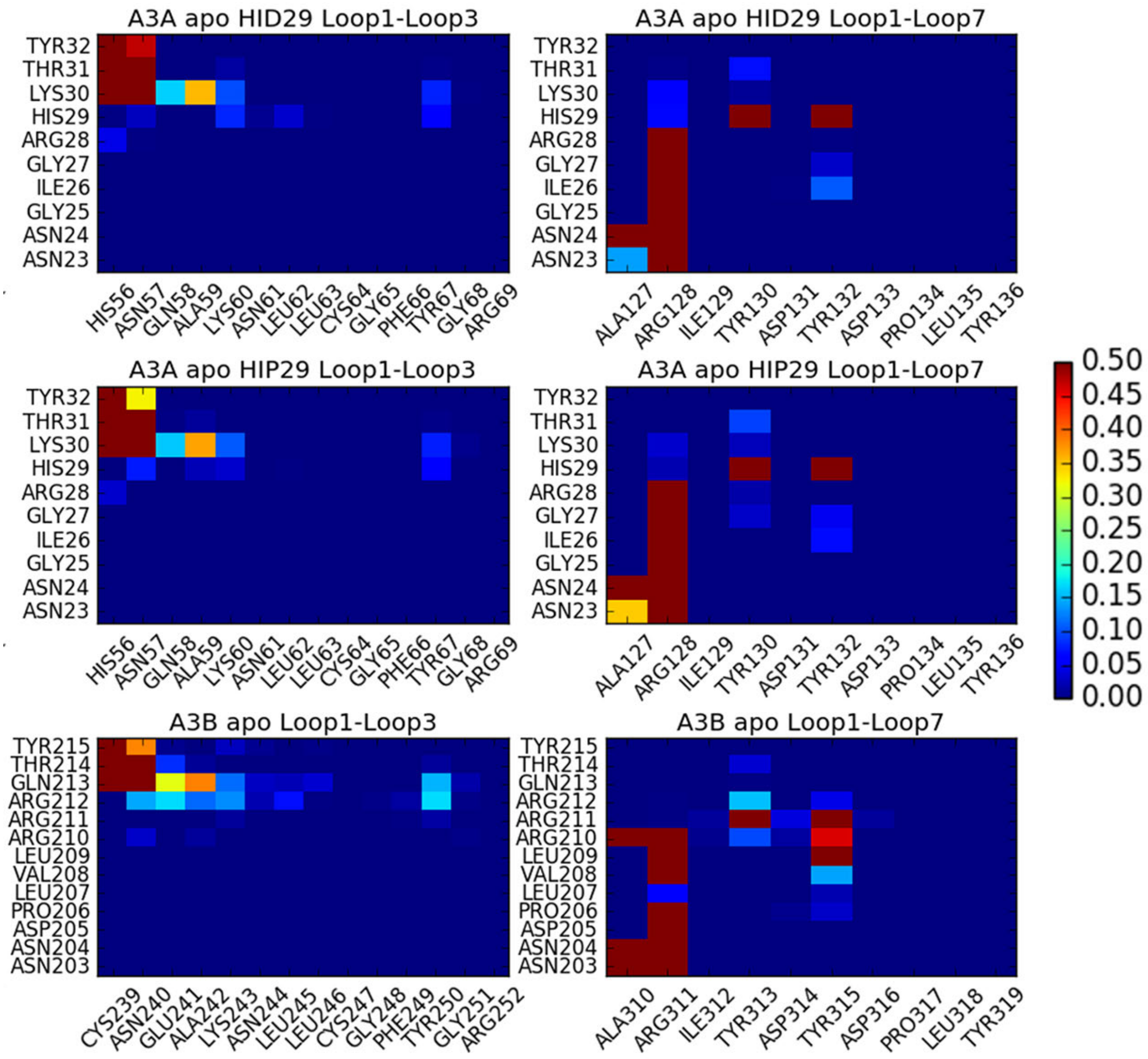

Fig. S7: Loop contacts in apo A3A and A3B simulations. Plots show the fraction of simulation snapshots in which residues are within 4 Å of each other (measured by their closest non-hydrogen atoms). The A3A simulations were run with both singly (HID) and doubly (HIP) protonated His29.

Alignment of APOBEC3B proteins from a few primates (CTD residues only)

```
                                                    Loop 1                              Loop 3
NP_001332901.1_Nomascus_leucogenys      MDPDTFTFNFNNDPLVFGRHQTYLCYEVERLDNGTWVKMDQHRGFLHNQAKDPLYGFDGRHTELC
XP_009232653.1_Pongo_abelii             MDPDTFTFNFNNDPFVLRRHQTYLCYEVEHLDNGTWVKMDQHRGSLHNQARNPLYGLDGRHAELR
NP_004891.4_Homo_sapiens                MDPDTFTFNFNNDPLVLRRRQTYLCYEVERLDNGTWVLMDQHMGFLCNEAKNLLCGFYGRHAELR
NP_001276568.1_Pan_troglodytes          MDPDTFTFNFNNDPLVLRRHQTYLCYEVERLDNGTWVLMDQHMGFLCNEAKNLLCGFYGRHAELR
NP_001288695.1_Pan_paniscus             MDPDTFTFNFNNDPLVLRRHQTYLCYEVERLDNGTWVLMDQHMGFLCNEAKNLLCGFYGRHAELR
XP_011887414.1_Cercocebus_atys          MDPDTFTFNFNNDLSVLGRHQTYLCYEVEHRDNGTWVPMDQHWGFLCNQARNPLHGVDSCHAELC
XP_011834236.1_Mandrillus_leucophaeus   MDPDTFTFNFNNDLSVLGRHQTYLCYEVERLDNGTWVPMDQHWGFLCNQARNPLHGVDSCHAELC
NP_001332930.1_Papio_anubis             MDPDTFTSNFNNDLSVRGRHQTYLCYEVEHLDNGTWVPMDQHWGFLCNQAKNLLHDDYGCHAELC
XP_005567303.1_Macaca_fascicularis      MDPDTFTSNFNNDLSVLGRHQTYLCYEVERLDNGTWVPMDQHXGFLCNQAKNVLRGDYGCHAELC
NP_001332883.1_Chlorocebus_sabaeus      MDPDTFTINFNNDLSVLGRRQTYLCYEVERLDNGTWVPMDQHWGFLCNQARNPLHGVDSCHVELC
NP_001233159.1_Macaca_mulatta           MDPDTFTSNFNNDLSVLGRHQTYLCYEVERLDNGTWVPMDQHWGFLCNQAKNVPRGDYGCHAELC
                                        ******* *****  *  *:*********: ****** **** * * *:*::   .  . *.**

                                                                                          Loop 7
NP_001332901.1_Nomascus_leucogenys      FLGLIPYWQLDLAQTYRVTWFISWSPCFSWGCAEQVRAFLQENTHMRLRIFAARIYDYDPLYKEA
XP_009232653.1_Pongo_abelii             FLGLLPYWQLDPAQIYRVTWFISWSPCFSWGCARQVRAFLQENTHVRLRIFAARIYDYDPLYKEA
NP_004891.4_Homo_sapiens                FLDLVPSLQLDPAQIYRVTWFISWSPCFSWGCAGEVRAFLQENTHVRLRIFAARIYDYDPLYKEA
NP_001276568.1_Pan_troglodytes          FLDLVPSLQLDPAQIYRVTWFISWSPCFSWGCAGQVRAFLQENTHVRLRIFAARIYDYDPLYKEA
NP_001288695.1_Pan_paniscus             FLDLVPSLQLDPAQIYRVTWFISWSPCFSWGCAGQVRAFLQENTHVRLRIFAARIYDYDPLYKEA
XP_011887414.1_Cercocebus_atys          FLDQVSSWQLDPAQTYRVTWFISWSPCFSWGCAEQVHAFLQENTHVRLRIFAARIYDYDPLYQEA
XP_011834236.1_Mandrillus_leucophaeus   FLDQVSSWQLDPAQTYRVTWFISWSPCFSWGCAEQVHAFLQENTHVRLRIFAARIYDYDPLYQEA
NP_001332930.1_Papio_anubis             FLGWVPSWQLDPAQMYRVTWFISWSPCFSGGCAEQVRAFLQENTHVRLRIFAARIYDYDPLYQEA
XP_005567303.1_Macaca_fascicularis      FLGWVPSWQLDPAQTYRVTWFISWSPCVSWGCAEQVRAFLQENTHVRLHIFAARIYDYDPLYQEA
NP_001332883.1_Chlorocebus_sabaeus      FLSQVSSWQLDPAQTYRVTWFISWSPCFSGGCAEQVRAFLQENTHVRLCIFAARIYNYDPLYQEA
NP_001233159.1_Macaca_mulatta           FLDQVSSWQLDPAQTYRVTWFISWSPCFSWGCADQVYAFLQENTHVRLRIFAARIYDYNPLYQEA
                                        **. :   *** ** ************.* *** :* ********:** *******:*:***:**

NP_001332901.1_Nomascus_leucogenys      LQMLRGAGAQVSIMTYDEFEHCWDTFVDHQGRPFQPWDGLEEHSQALSGRLQAILLNQGN
XP_009232653.1_Pongo_abelii             LQMLRDAGAQVSIMTYDEFEYCWNTFVDHQGCPFQPWDGLEEHSQALSGKLQAILLNQGN
NP_004891.4_Homo_sapiens                LQMLRDAGAQVSIMTYDEFEYCWDTFVYRQGCPFQPWDGLEEHSQALSGRLRAILQNQGN
NP_001276568.1_Pan_troglodytes          LQMLRDAGAQVSIMTYDEFEYCWDTFVYRQGCPFQPWDGLEEHSQALSGRLRAILQNQGN
NP_001288695.1_Pan_paniscus             LQMLRDAGAQVSIMTYDEFEYCWDTFVYRQGCPFQPWDGLEEHSQALSGRLRAILQNQGN
XP_011887414.1_Cercocebus_atys          LRTLQDAGAQVSIMTYEEFEYCWDTFVDRQGCPFQPWDGLDEHSQALSGRLQAILQGN--
XP_011834236.1_Mandrillus_leucophaeus   LRTLRDAGAQVFIMTYKEFEYCWDTFVDRQGRPFQPWDGLDEHSQALSGRLQAILQNQGN
NP_001332930.1_Papio_anubis             LRMLRDAGAQVSIMTYEEFEYCWDTFVDRQGCPFQPWDGLDEHSQALSGRLRAILQNQGN
XP_005567303.1_Macaca_fascicularis      LRTLRDAGAQVSIMTYDEFEYCWDTFVDRQGRPFQPWDGLDEHSQALSGRLRAILQNQGN
NP_001332883.1_Chlorocebus_sabaeus      LRMLRDAGAQVSIMTYEEFEYCWDTFVDRQGCPFQPWDGLDEHSQALSGRLRAILQNQGN
NP_001233159.1_Macaca_mulatta           LRTLRDAGAQVSIMTYDEFEYCWDTFVDRQGRPFQPWDGLDEHSQALSGRLRAILQNQGN
                                        *: *:.***** ****.***:**:*** :** ********:********:*:*** .:
```

Fig. S8: Amino acid sequence alignment of primate A3Bctd.

Loops 1, 3, 7 surrounding the active site are highlighted.

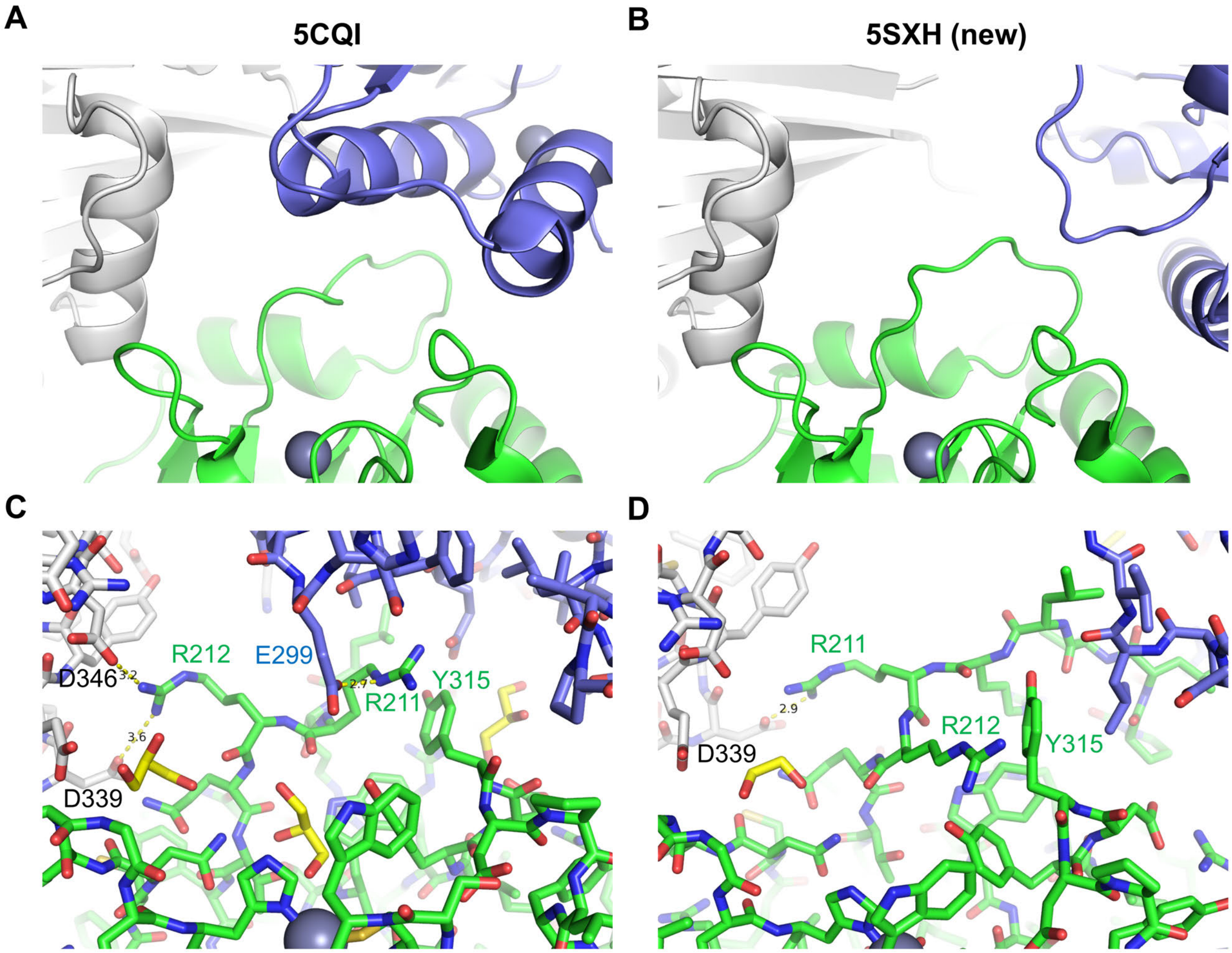

Fig. S9: Crystal lattice contacts around loop 1.

Crystal packing interactions involve a flipped arginine side chain, both in the previously reported (A, C) and the new (B, D) crystal form.

Legends for Supplementary videos

Video 1: Dynamics of apo form of wild-type A3B observed in MD copy1.

Wild-type A3Bctd is depicted in silver-colored ribbons with Y315, W281, R210, R211, R212 and R313 in sticks colored according to atom types (N atoms in blue, C atoms in cyan, O atoms in red). Hydrogen atoms and water molecules are not shown for simplicity. A computational model of DNA-bound wild-type A3B, generated based on the DNA-bound A3Bctd variant crystal structure (with loop1 from A3A) and by restoring the natural loop1 of A3B, is shown in orange-colored ribbons with the residues mentioned above in orange-colored sticks. The simulation is 1 μs-long.

Video 2: Dynamics of apo form of wild-type A3A observed in MD copy1 (HIP29)

Wild-type A3A is depicted in silver-colored ribbons with Y132, W98, R28, H29, K30 and R130 in sticks colored according to atom types (N atoms in blue, C atoms in cyan, O atoms in red). Hydrogen atoms and water molecules are not shown for simplicity. The wild-type model of DNA-bound A3A generated using DNA-bound A3A crystal structure is shown in orange-colored ribbons with the residues mentioned above in orange-colored sticks. The simulation is 1 μs-long. This movie contains 'smoothing' which in some instances provides a non-physical depiction of residue side chains (due to positional averaging among the frames for smooth visualization).
