# Supplementary figures and images for: Conformational Switch Regulates the DNA Cytosine Deaminase Activity of Human APOBEC3B
Source: Sci Rep. 2017 Dec 12;7:17415. doi: 10.1038/s41598-017-17694-3 (PMC5727031; doi:10.1038/s41598-017-17694-3)

Figure 6

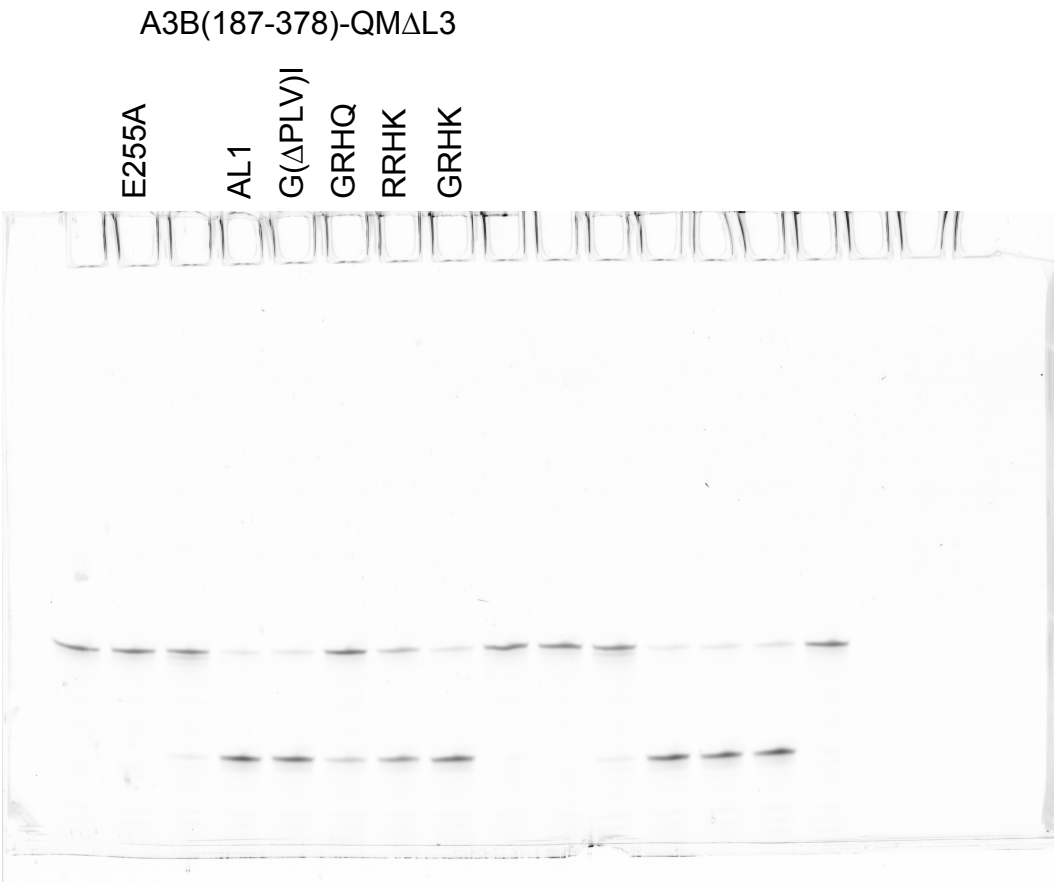

Figure S5A

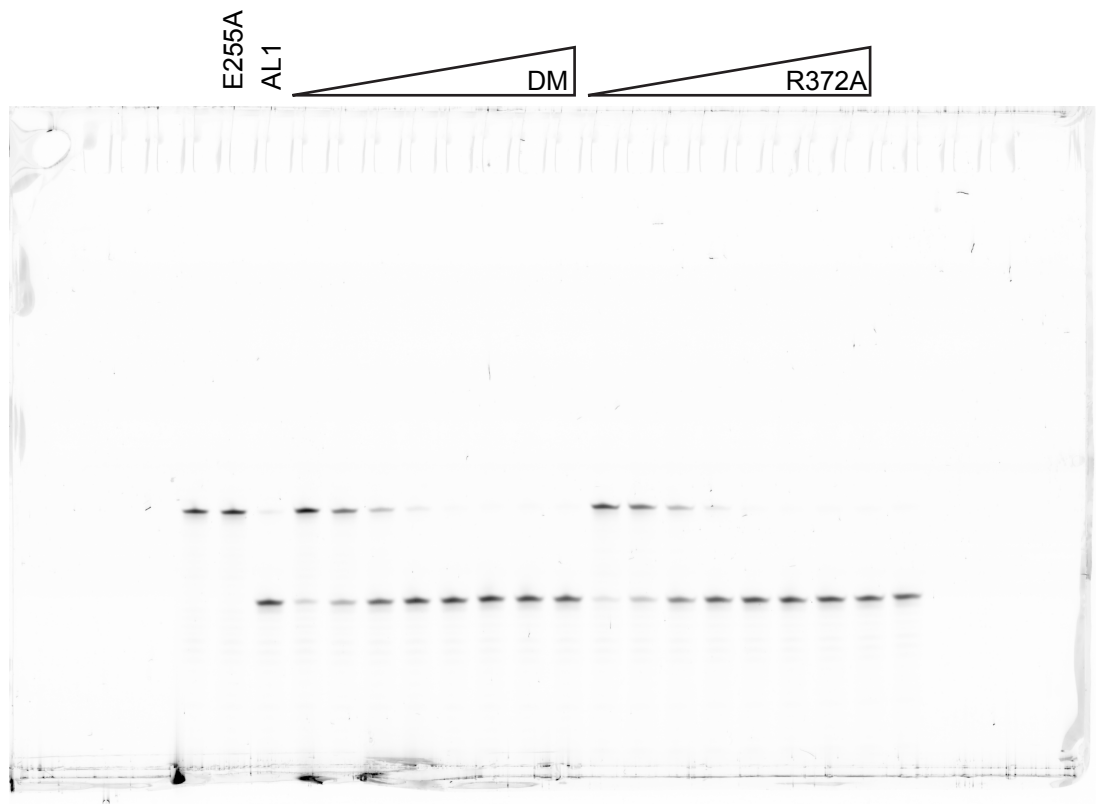

Supplement: Supplementary file 4 — Uncropped gel images [file 41598_2017_17694_MOESM4_ESM.pdf]
